# Pathogenic and Opportunistic *Vibrio* spp. Associated with Vibriosis Incidences in the Greek Aquaculture: The Role of *Vibrio harveyi* as the Principal Cause of Vibriosis

**DOI:** 10.3390/microorganisms11051197

**Published:** 2023-05-04

**Authors:** Adriana Triga, Maria Smyrli, Pantelis Katharios

**Affiliations:** 1 Institute of Marine Biology, Biotechnology and Aquaculture (IMBBC), Hellenic Centre for Marine Research (HCMR), P.O. Box 2214, 71500 Heraklion, Greece; triga@hcmr.gr (A.T.); msmyrli@hcmr.gr (M.S.); 2 Department of Biology, University of Crete, P.O. Box 1470, 71110 Heraklion, Greece

**Keywords:** vibriosis, *Vibrio harveyi*, European seabass, *toxR*, metabolic fingerprint

## Abstract

A monitoring program to follow vibriosis incidents in the Greek marine aquaculture was implemented over the past 13 years. 273 isolates, from various cases originating from eight regions and nine hosts, were collected and characterized. The main aquaculture species of the survey were the European seabass (*Dicentrarchus labrax*) and the gilthead seabream (*Sparus aurata*). Various species of *Vibrionaceae* were associated with vibriosis. *Vibrio harveyi* had the highest prevalence and was isolated throughout the year from all hosts. During the warm months, *Vibrio harveyi* prevailed with frequent co-isolations of *Photobacterium damselae* subsp. *damselae* and *Vibrio alginolyticus*, while during spring, other *Vibrio* species were more abundant, such as *Vibrio lentus, Vibrio cyclitrophicus,* and *Vibrio gigantis.* Phylogenetic analysis using the *mreB* gene and the metabolic fingerprint of the isolates showed great variability within the species of the collection. The severity of the disease and the frequency of outbreaks make vibriosis (that is, mainly attributed to *V. harveyi*) an important concern for the regional aquaculture sector.

## 1. Introduction

Vibriosis is one of the earliest infectious diseases described in fish [1]. It is by far the most significant disease affecting marine aquaculture and it is responsible for huge economic losses of the industry. Taking together fish and shrimp aquaculture, financial damage because of vibriosis exceeds 1 billion USD per year [2]. Originally, vibriosis in fish of the Mediterranean marine aquaculture was attributed almost exclusively to *Vibrio anguillarum*, but due to the success of commercially available vaccines, this species is no longer a commonly isolated causative agent of the disease. Instead, several other species of the genus have emerged as serious fish pathogens and taken up the niche of *V. anguillarum*. These include *V. harveyi*, *V. alginolyticus*, and *V. splendidus*, but also *Photobacterium damselae* subsp. *damselae* (formerly *Vibrio damsela*), which is a member of *Vibrionaceae* as well [3,4,5,6]. 

The diversity of the vibrios and the complexity of their phylogeny, especially of the Harveyi and the Splendidus clades, which include several opportunistic pathogens for fish and marine animals [4,7,8,9,10], suggest that vibriosis is a multifaceted disease, especially regarding the causative agents. Recent advances in genomic science have revealed many novel species of the genus, which were initially impossible to differentiate using routine diagnostics but that have also helped to identify their wide arsenal of virulence factors, thus classifying them as presumptive pathogens [9,11,12]. On the other hand, most studies regarding novel species of *Vibrio* lack reliable in vivo pathogenicity testing, and, therefore, the actual role of several species as real pathogens is somewhat obscured. Moreover, many different species of *Vibrio* can be found co-infecting the same host or they can be simultaneously present in Vibriosis outbreaks.

In Greece, which is among the main producers of gilthead seabream (*Sparus aurata*) and European seabass (*Dicentrarchus labrax*), vibriosis has been regarded as the most serious disease of these species [13,14]. This is also the case for other fish species cultured in the Mediterranean Sea [15]. Even though there have been occasional reports in the scientific literature of vibriosis in Greek aquaculture, the only systematic attempt to provide data on this issue was for the period 1998–2013, with a focus on European seabass [13]. Here, we have investigated incidents of vibriosis in several locations in Greece, including several commercial fish farms, providing data on their phenotypic and genetic profile. For comparative purposes, we included isolates from two hosts commonly found in aquaculture sites in the Red Sea, the gilthead seabream and the Nile tilapia. The former is a frequent host in our study. Additionally, we obtained *Vibrio* isolates from various hosts, including wild fish hosted in Cretaquarium, a public aquarium of HCMR in Crete that houses common aquaculture species with others found in the region. The aim of the study was to investigate the diversity of the vibrios, which are routinely isolated during vibriosis in Greece, with a special focus on *Vibrio harveyi*, and to discuss their role as opportunistic pathogens.

## 2. Materials and Methods

### 2.1. Sampling

**Figure 1:**
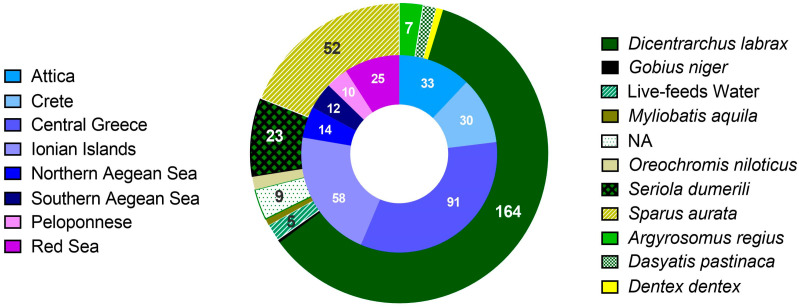
The total number of 273 isolates divided according to the isolation region (inner ring), and the host (outer ring), groups with less than 5 isolates are not annotated, NA: not applicable.

The strains of the bacterial collection analyzed herein have been isolated during monitoring samplings and seasonal outbreaks of vibriosis between 2007–2021, with emphasis on *V. harveyi*. The most intensive sampling effort took place during the final three years. Sampling covered 7 regions of Greece with 29 locations, and one in the Red Sea (Figure 1). Most *Vibrio* isolates included in the study were isolated from European seabass followed by gilthead seabream, which are the main fish species reared in the Mediterranean Sea. Other hosts included novel aquaculture species for the Mediterranean, such as the greater amberjack (*Seriola dumerili*), the meagre (*Argyrosomus regius*), and the common dentex (*Dentex dentex*). Additionally, samples from gilthead seabream and Nile tilapia (reared in seawater) from Yanbu, in the Red Sea, were also included for the purpose of comparative analysis. We have also included isolates from fish caught from the wild and kept in Cretaquarium, the public aquarium of HCMR in Crete, Greece, such as the common eagle ray (*Myliobatis aquila*), the common stingray (*Dasyatis pastinaca*), and the black goby (*Gobius niger*). Furthermore, isolates from the water of hatchery facilities have been included in the study (Figure 1). Freshly dead, moribund, and apparently healthy fish from all rearing stages were examined. Bacteria were isolated mainly from the kidney, but, in some cases, bacteria from the spleen, intestine, skin, brain, ascetic fluid, and gills were also sampled using standard aseptic isolation techniques. Water samples were filtered through a 0.22 μm sterile polycarbonate filter, which was plated in microbiological media (see below). 

The initial bacterial isolation was mainly done using the general medium, tryptone soy agar (TSA) (Neogen Culture Media, Heywood, UK), supplemented with 2% NaCl and the *Vibrio*-selective medium thiosulfate-citrate-bile salts-sucrose agar (TCBS) (Neogen Culture Media, Heywood, UK) [16]. Plates were incubated in 25 °C, then colonies were purified by more than one round of re-streaking on TSA plates and stored in −80 °C Microbank tubes (Pro-lab Diagnostics, Richmond Hill, ON, Canada).

### 2.2. Histopathology

Tissue samples from fish exhibiting clinical signs of vibriosis were preserved in 10% neutral buffered formalin for histological examination. Formalin-fixed tissues were dehydrated in an ascending 70–96% ethanol series and embedded in glycol methacrylate resin (Technovit 7100, Heraeus Kulzer, Hanau, Germany). The RM2245 microtome (Leica, Humbloch, Germany) with disposable blades was used to cut serial sections (3–5 μm) and the dry slides were stained with a polychrome stain (methylene blue/azure II/basic fuchsin).

### 2.3. Molecular Identification

Identification of the isolates was based on molecular methods. DNA extraction was performed following boiling method. Briefly, a colony from an overnight culture was suspended in molecular biology grade water (Fisher Scientific, Geel, Belgium), boiled for 10 min, cooled for 4 min, and following centrifugation, the supernatant was collected and stored at −20 °C until use. Initially, all samples (n = 273) were screened using PCR with *toxR* primers targeting the 390-bp fragment of the *V. harveyi toxR* locus [17] to detect *V. harveyi* in the samples of the collection. Then, selected isolates, both *toxR+* and *toxR−* were further analyzed with PCR using the 16S rRNA bacterial universal primers and *mreB* primers [18] (Table S1). The *mreB* locus provides reliable identification for vibrios of the Harveyi clade with higher discrimination resolution than 16S [19,20]. PCR products of the two loci selected for sequencing were purified with the commercial Qiaquick PCR purification kit (Qiagen, Hilden, Germany). Sanger sequencing was conducted using ABI3730xl DNA analyzer (CEMIA SA, Larissa, Greece). Electropherograms were analyzed and the sequences were aligned in the Geneious Prime 11.0.14 (www.geneious.com, accessed 30 April 2023), using MUSCLE [21]. The sequences were identified with the BLASTn similarity search algorithm [22] in GenBank (National Centre for Biotechnology Information, Bethesda, MD, USA). The usability of the *toxR* PCR assay as a reliable and rapid diagnostic tool for *Vibrio harveyi* was evaluated by calculating the accuracy, specificity, and sensitivity ratios according to the following equations [23]

$$\mathrm{Accuracy}\;(\%)=\frac{TP+TN}{TP+TN+FP+FN}\times 100$$

$$\mathrm{Sensitivity}\;(\%)=\frac{TP}{TP+FN}\times 100$$

$$\mathrm{Specificity}\;(\%)=\frac{TN}{TN+FP}\times 100$$

where, *TP*: true positive, *TN*: true negative, *FP*: false positive, *FN*: false negative

The phylogenetic tree for the *mreB* was constructed using the Maximum-likelihood (ML) method and Tamura-Nei model, the bootstrap consensus tree was inferred from 500 replicates, and the analysis was conducted in MEGA X [24].

### 2.4. Metabolic Fingerprint

The GEN III MicroPlate (BIOLOG, Hayward, CA, USA) was used for the biochemical characterization of 96 isolates, 72 of which had been molecularly identified as *V. harveyi*. The results of the GEN III MicroPlate reactions for carbon utilization and chemical resistance were read after 48 h incubation at 25 °C and were also used for identification to genus level. Unsupervised hierarchical cluster analysis was done using squared Euclidean distance measure and Ward linkage with IBM SPSS Statistics 21. This analysis was done to identify groups of isolates based on the metabolic attributes of their phenotype, and a heatmap was generated using the GraphPad Prism 9 (GraphPad Software, San Diego, CA, USA).

## 3. Results

### 3.1. Bacterial Identification

Of the 273 isolates, 143 were positive in the *toxR* PCR and were presumptively identified as *Vibrio harveyi*. The definitive identification of the bacteria of the collection was based on the combination of the results obtained from *mreB* and 16S sequencing performed for 80 of the *toxR* positive strains and for all *toxR* negative strains (n = 130).

The PCR targeting the *toxR* gene was accurate in identifying *V. harveyi* following confirmation of the sequencing results with Blast in the Genbank nr database, as 78 out of the 80 sequenced strains were *V. harveyi*. One strain was identified as *Photobacterium damselae* subsp. *damselae* and one as *Vibrio* sp. with *Vibrio owensii* being the most similar taxon, according to the *mreB* sequencing. Of the remaining 130 strains, which were *toxR*-, only three were identified as *Vibrio harveyi*. According to the sequencing results, the toxR PCR assay was 97.6% accurate, 96.3% sensitive, and 98.4% specific. All sequencies obtained have been deposited in NCBI. The results of the identification and accession numbers are provided in Table S2.

### 3.2. Seasonality of Vibrio Species and Phylogenetic Relationships

**Figure 2:**
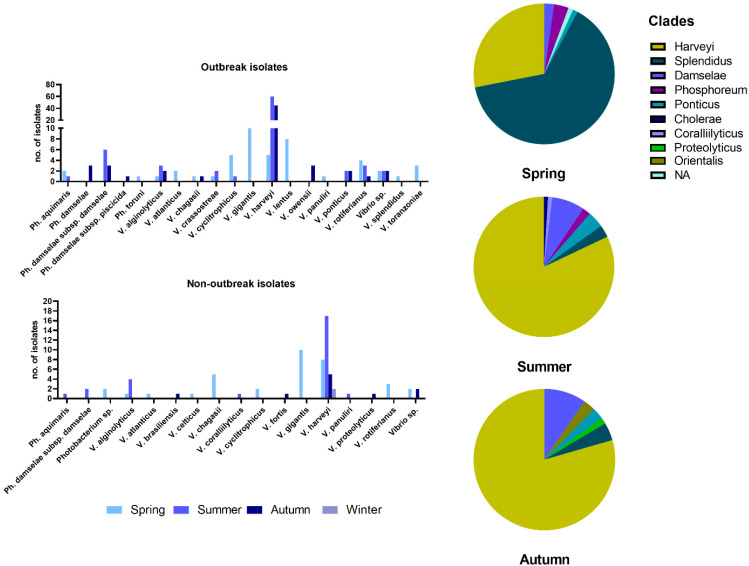
Prevalence of bacterial species identified during and between outbreaks (bar charts on the left) and seasonal representation of the taxonomical clades identified (pie charts on the right).

The most commonly found species in all samplings was *V. harveyi*, followed by *V. gigantis*, *V. rotiferianus*, and *V. alginolyticus* (Figure 2). Within the samplings during vibriosis outbreaks but also within the non-outbreak samplings, it was evident that the *V. harveyi* was prevailing with the rise of temperature. However, during April and May, *V. chagasii*, *V. cyclitrophicus*, *V. gigantis*, *V. lentus*, and *V. rotiferianus* were the common findings. The species *V. gigantis* and *V. cyclitrophicus*, in particular, were isolated only during spring months. Bacteria of the *Photobacterium* genus represented by 6 taxa were frequently isolated from diseased fish, with *Ph. damselae* subsp. *damselae* being the most prevalent. In these cases, *Photobacterium* spp. were always co-isolated with various *Vibrio* spp., such as *V. harveyi*.

The main sampling effort was made for the outbreak samplings, where more bacteria were isolated (n = 200), stored, and analyzed to create a strain base for the study of the disease. These strains were mainly isolated during summer and autumn months, and most were *V. harveyi*. Bacteria of the species *V. gigantis* and *V. lentus* were found more often than *V. harveyi* during the spring outbreaks. In two cases, *V. harveyi* was co-isolated with bacteria of the genus *Aeromonas*, and at least in one of these cases, the principal pathogen was *Aeromonas veronii*, based on the clinical picture of the fish. In the group of non-outbreak samplings, 73 isolates were collected, and the most common were *V. gigantis* in spring and *V. harveyi* in summer and autumn.

Taken together, we observed that in the few springtime outbreaks, bacteria of the Splendidus clade were isolated more frequently (Figure 2), while in summer, bacteria of the Harveyi clade prevailed. In autumn, the 15 cases studied were dominated by *V. harveyi*, and some of them were ongoing outbreaks that had started in summer. Bacteria of the Damselae clade were also isolated during summer and autumn months as co-infecting agents with *V. harveyi*.

**Figure 3:**
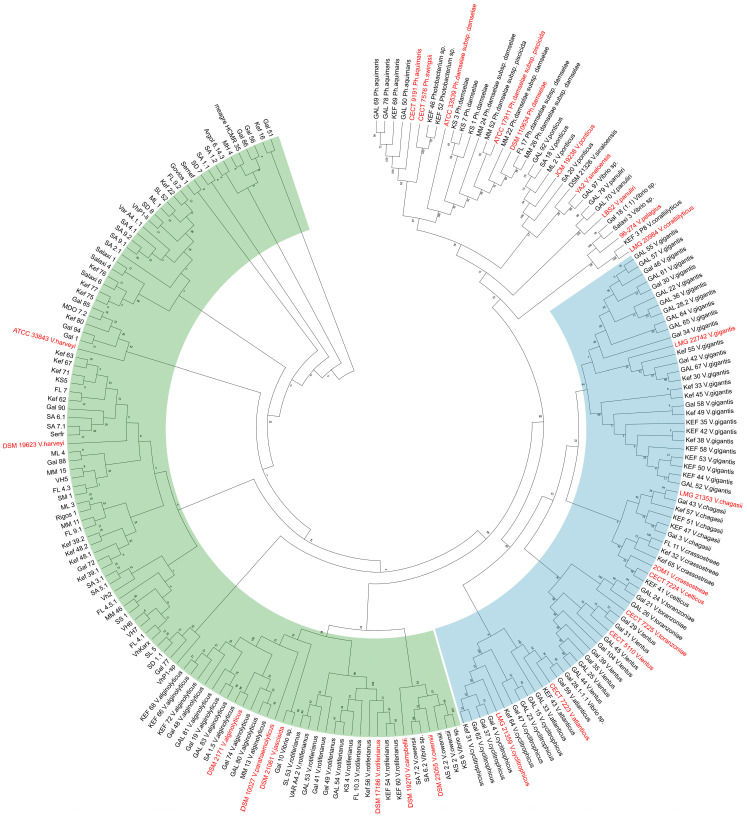
Unrooted Maximum-Likelihood tree of 211 nt sequences, branches corresponding to partitions reproduced in less than 50% bootstrap replicates are collapsed. The percentage of replicate trees in which the associated taxa clustered together in the bootstrap test (500 replicates) are shown next to the branches. *V. harveyi* isolates appear only by codename, and Genbank sequences are red. Vibrio clades are colored: Harveyi (green) and Splendidus (blue).

A maximum-likelihood tree was constructed using 211 *mreB* sequences (658-bp fragment) of isolates of the study (n = 184) and publicly available ones from the NCBI Genbank database (n = 27) (Table S3). The species of the Harveyi clade and the species of the Splendidus clade are grouped separately (Figure 3), in detail, the identified species that belong to the Harveyi clade are *V. harveyi, V. alginolyticus, V. rotiferianus,* and *V. owensii*, and the species of the Splendidus clade were *V. cyclitrophicus, V. atlanticus, V. lentus, V. toranzoniae, V. crassostreae, V. chagasii*, and *V. gigantis.* In the ML tree, there were also some reference strains added of species of the Harveyi clade, such as *V. parahaemolyticus, V. jascicida*, and *V. cambelii*. According to the *mreB* ML tree, *V. harveyi* formed close monophyletic genetic groups with many genetic lineages, as also observed, albeit to a lesser degree, for the other species. The clustered isolates do not reflect chronological order or location, and isolates from the aquarium and the Red Sea do not form separate groups.

### 3.3. Metabolic Fingerprint

The 94 variables of the Gen III metabolic fingerprint revealed a great plasticity within and between the species. The *V. harveyi* strains were all growing in the NaCl gradient tested (1–8% NaCl), 83% fermented sucrose, 33% D-salicin, 85% D-cellubiose, 81% D-galactose, 42% utilized L-arginine, and almost none utilized myo-inositol, α-D-lactose, and D-arabitol (Table 1). All of them showed growth at pH = 6 but a 14% at pH = 5. The strains were resistant to 2% Sodium Lactate, Fusidic Acid, Niaproof 4, Rifamycin SV, and Tetrazolium Blue, and were sensitive to Minocycline and Nalidixic Acid. 

**Figure 4:**
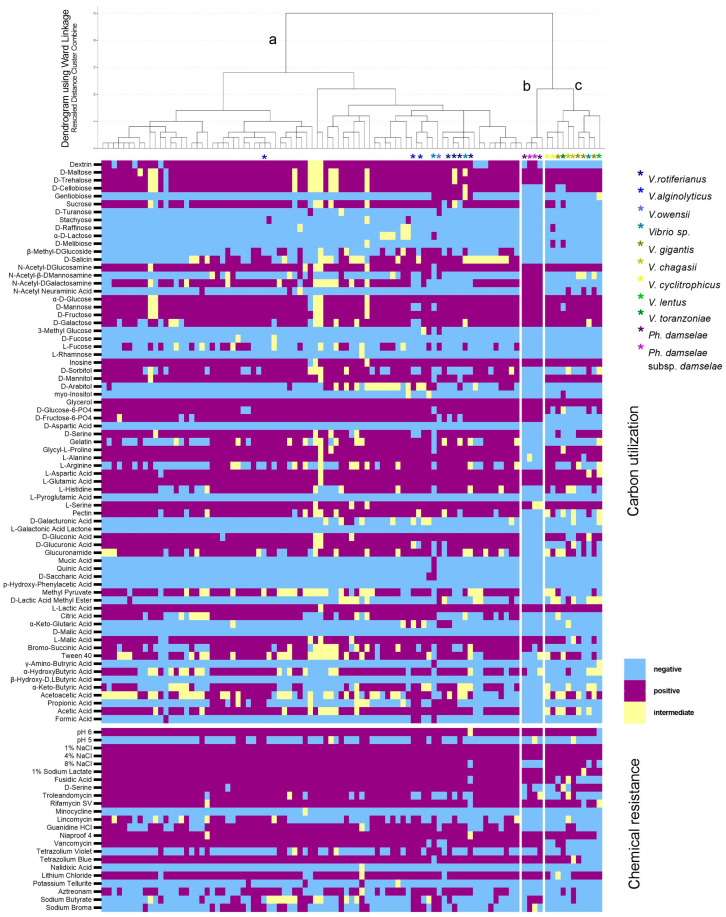
Results of the Gen III MicroPlate reactions of 72 V. harveyi and 24 other *Vibrio* spp. and *Photobacterium* spp. isolates, and isolates other than *V. harveyi* are defined with an asterisk. The hierarchical clustering dendrogram based on the 94 reactions is shown at the top (a: Harveyi clade, b: *Photobacterium* spp., c: Splendidus clade). The heatmap of the carbon utilization reactions (upper) and chemical resistance reactions (bottom) for all isolates tested is shown below the dendrogram.

The hierarchical clustering resulted in the classification of 96 clinical isolates of 7 different species according to their metabolic fingerprint into 3 clusters (Figure 4). Cluster A included the Harveyi clade isolates, which were the majority, and it was separated from clusters B and C that comprised the *Photobacterium* isolates and the Splendidus clade, respectively. Once again, the clustering of the strains did not reflect their source. Reactions that were quite variable for cluster A but negative for clusters B and C were the utilization of L-Fucose, D-Salicin, and Citric Acid (Figure 4). L-Arginine, Bromosucchinic Acid, a-Ketobutyric Acid, Acetoacetic Acid, D-Sorbitol utilization, and Aztreonam and Sodium Butyrate resistance reactions were variable in all clusters. *P. damselae* subsp. *damselae*, unlike other isolates, were negative in the utilization of D-Mannitol, Pectin, D-Gluconic, D-Glucuronic Acid, and Glucoronamide, and they did not grow at 8% NaCl.

### 3.4. Pathology of Vibriosis

**Figure 5:**
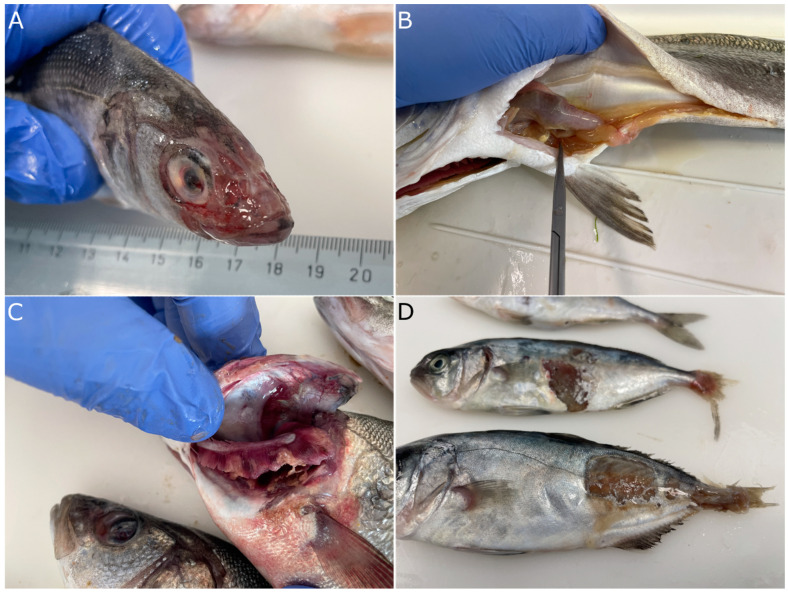
Typical clinical picture of fish affected by *Vibrio harveyi*. (**A**). European seabass with eroded skin at the area of the head and ophthalmitis. (**B**). Catarrhal enteritis in European seabass. (**C**). Focal necrosis of the gills of seabass (note the eye lesion of the second fish in the picture). (**D**). Severe dermal lesions of greater amberjack and caudal fin erosion.

Vibriosis caused by *V. harveyi* is characterized by epidermal lesions that range from superficial to deep depending on the species affected (Figure 5A,D). In progressed cases, external hemorrhages and petechia are evident mostly in the area behind the operculum, at the basis of the fins and in the abdomen. In the European seabass, the area of the head is eroded (Figure 5A) and appears white when the fish are still in the water. In many cases, the bacteria affect the eyes, causing opaqueness of the cornea and consequently blindness (Figure 5A,C). Focal gill necrosis is also common during outbreaks, especially in the summer (Figure 5C). Internally, the most prominent clinical sign of the disease is extensive catarrhal enteritis (Figure 5B). As the disease progresses, it becomes systemic and bacteria are disseminated in other organs like the spleen, liver, and brain, which often appear inflamed. 

**Figure 6:**
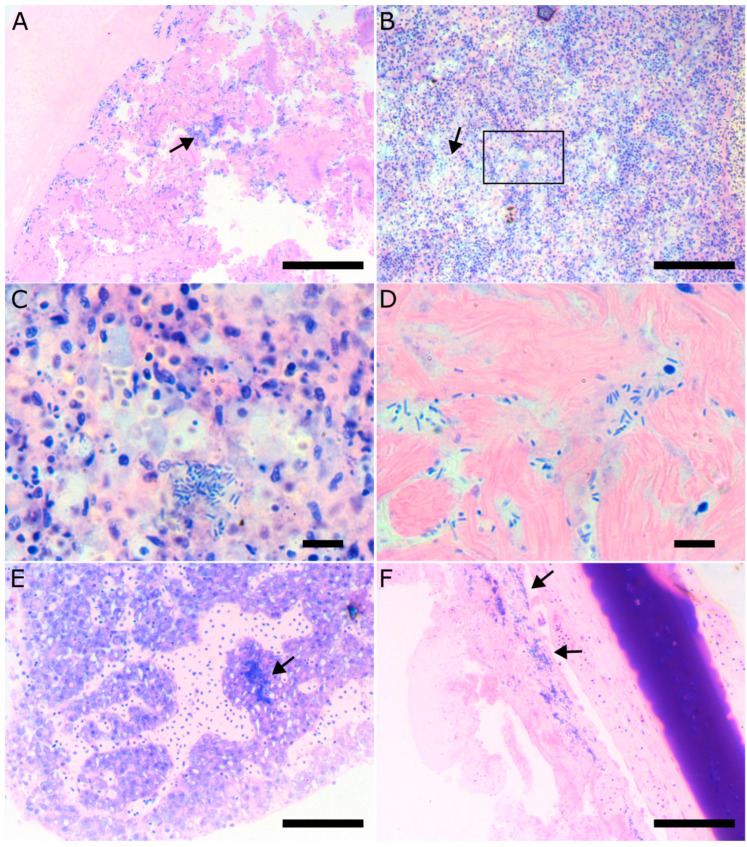
Histopathology of European seabass affected by *Vibrio harveyi*. (**A**). Histological section of the intestine. Note the destruction of the tissue architecture, the loss of intestinal villi, and the extensive colonization of the tissue by bacteria stained blue (arrow). (**B**). Focal necrosis of the spleen (arrow). (**C**). Higher magnification of the area indicated by the rectangular in B. Bacterial colonies are associated with the necrotic areas. (**D**). Bacteria in the ventricular myocardium. (**E**). Bacterial colony (arrow) in the liver. (**F**). Bacterial colonies in the corneal stroma of the eye.

Histologically, fish affected by vibriosis caused by *V. harveyi* displayed severe pathology of the intestine with complete destruction of the normal tissue architecture, loss of the intestinal villi, and extreme colonization of the remaining tissue by bacteria (Figure 6A). Focal areas of necrosis could be found in the spleen associated with bacterial colonies (Figure 6B,C). As the disease progresses and becomes systemic, bacterial colonies can be found in other tissue like the liver, the heart, the eye, and the brain (Figure 6D–F).

## 4. Discussion

This study represents an extensive effort to characterize vibriosis in the Eastern Mediterranean aquaculture and to collect bacterial isolates associated with the disease. Our results showed that *V. harveyi* is the main pathogen associated with vibriosis during summer and autumn, but other species mainly of the Splendidus clade such as *V. gigantis, V. lentus, V. cyclitrophicus*, and *V. chagasii* occurred frequently in spring. The disease affects mainly the European seabass, but other fish species grown in the area are also affected. Temperature has been considered as the most important risk factor [13]. Elevated temperatures affect *V. harveyi* in many ways. Its metabolic pathways and fitness become impaired, while virulence factors such as chemotaxis proteins and secretion system components become upregulated [25]. Adhesion ability of the species has been reported to peak close to 30 °C [26]. These attributes explain the upsurge of pathogenic isolates during summer. Moreover, fish immune system and stress coping mechanisms are also compromised in higher temperatures [14,27]. Although the highest number of isolates were obtained in summer, the diversity of the vibrios was higher in spring. A similar observation was made for *Vibrio* spp. in the aquaculture zone of Dongshan Bay in China [28]. In our study, outbreaks occurring during spring when water temperature is much lower (13–17 °C), and were associated with bacteria of the Splendidus clade. High mortalities of *D. dentex* during October to December were attributed to *V. splendidus* which was obtained in pure cultures from kidneys and ulcers of moribund fish [29]. Our results agree with the study of Bellos et al. (2015), conducted also in Greece, where during low water temperature, more cases of vibriosis were caused by *V. splendidus* and *V. alginolyticus*. Furthermore, *V. gigantis* and *V. cyclitrophicus*, also members of the Splendidus clade, were detected more often in colder months. Both species have been associated with invertebrate aquaculture, and although they were regarded as non-pathogenic or having low virulence to aquatic animals [30], their actual role has not yet been elucidated. It is noteworthy that the isolation of *V. gigantis* has been reported to peak during early spring in cultured olive flounder (*Paralichthys olivaceus*) farming sites in Jeju Island [31], before pathogenic species start to be detected more.

In the Mediterranean Sea, *V. harveyi* has been isolated from all stages of rearing, regardless of the age of the fish [32,33,34]. Many virulent strains were found in a gilthead seabream hatchery in Malta, causing morbidity and mortalities [35]. The species, along with other opportunistic pathogens, becomes part of the microbiota when fish are introduced to live feeds [36,37] or through the rearing tanks and water inlets [34]. In our study, very few *V. harveyi* strains were isolated from the hatchery environment (tanks, live feeds) and only in two cases was this species related to disease outbreak. On the other hand, the pathogen is more relevant to fish up to 50 g in weight, and most outbreaks are recorded during the first summer of the fish in the cages. This is in agreement with the observations made in a barramundi (*Lates calcarifer*) aquaculture in Vietnam, where *Vibrio harveyi* is associated with mortalities mostly in younger fish after their transfer to sea cages [38].

The clinical signs and the pathology caused by *Vibrio harveyi* recorded in the current study agrees with previous reports [32]. The principal lesions include superficial ulcers of the epidermis and fin erosion, hemorrhages, and severe catarrhal enteritis. The presence of lesions in the area of the head and ophthalmitis are more conspicuous in European seabass. The clinical picture of the fish resembles tenacibaculosis caused by *Tenacibaculum maritimum* (*T. mar*) [39], but in these cases, the morphology of the bacterial colonies on the gills and the skin of the affected fish together with the elongated form of the *T. mar* bacteria can help significantly in the differentiation of the diseases. 

Many different fish species are affected by *V. harveyi*, with prominent cases of this being the European seabass and the gilthead seabream, with the first being more sensitive to the pathogen than the last [40]. Cultured *D. dentex* has also been reported to be prone to vibriosis in Spain, with mortalities exceeding 50% caused by combined parasitic and bacterial infections of *V. alginolyticus, V. splendidus*, and *P. damselae* [41]. Another novel fish species for the Mediterranean aquaculture, the greater amberjack, has been reported to be affected by vibriosis caused by *V. harveyi* [42]. Furthermore, we have included *V. harveyi* strains obtained from Nile tilapia suffering from vibriosis when cultured in seawater in the Red Sea. To our knowledge, this is one of the few incidences of disease caused by *V. harveyi* in this species, although its presence as part of this fish microbiome has been reported previously [43]. Various vibrios, including *V. harveyi* and *V. alginolyticus*, are normal constituents of its microbiome [44], and, therefore, their pathogenic action is probably triggered by other environmental factors and facilitated by a suboptimal immune status of the fish host. 

Wild marine fish populations are carriers of vibrios as well, and vibriosis has been reported in wild fish populations previously [45]. Studies including both farmed and wild fish in the Mediterranean are rare, and they are mostly focused on the presence of vibrios in wild caught fish. A relatively low prevalence of *Vibrio* spp. ~7% of total isolates was mentioned in Israeli coast, sampling from farmed gilthead seabream, Mediterranean indigenous, striped-red mullet (*Mullus surmuletus*), round sardinella (*Sardinella aurita*), Lessepsian Randall’s threadfin bream (*Nemipterus randalli*), and lizardfish (*Saurida lessepsianus*) [46]. The same prevalence was also reported on the Algerian coast when wild European seabass and gilthead seabream were sampled [47]. Pathogen interaction between wild and cultured fish is an ongoing concern that originally started out with sea lice in salmon aquaculture. However, it is highly likely that fish populations reared in sea cages are being infected by wild fish, which are asymptomatic carriers of the pathogens and then become a reservoir for their propagation due to the stressful condition of the hosts caused by the rearing process. This kind of interaction, although not experimentally demonstrated, has long been suspected for vibriosis, too [48]. 

Co-infections by *Vibrio* spp. and other bacterial pathogens were common findings of the current study, but they have also been reported elsewhere [32]. It has been reported that co-infection of the hybrid grouper (*E. polyphekadion* × *E. fuscoguttatus*) by *V. harveyi* and *V. alginolyticus* has resulted in more severe symptoms and mortalities compared to those observed in single infections [49]. Moreover, co-infections with pathogens other than vibrios are not uncommon in Mediterranean aquaculture [14]. For example, gilthead seabream and European seabass are affected by the gill monogenean parasites, *Sparicotyle chrysophrii* and *Diplectanum aequans*, respectively, which may act additively on the severity of the outbreaks. Other parasites causing similar problems but not as widely prevalent as the former include *Lernanthropus croyeri* (Copepoda) and *Caligus* spp. (Isopoda). Finally, epitheliocystis disease, which is a gill pathology associated with intracellular bacteria (beta-, gammaproteobacterial and Chlamydia), may affect gill function and could also be a contributing factor to the severity of vibriosis when it is currently present in the outbreaks.

Other Harveyi clade species have also been often discovered from diseased and healthy fish. Outbreaks in gilthead seabream farming in Southwestern Spain recorded during cold months had higher isolation rates of *V. alginolyticus* followed by unidentified vibrios and *V. harveyi* [50]. In Eastern Adriatic aquaculture settings, *V. alginolyticus* was the only species isolated with higher frequency in spring during non-outbreak samplings from European seabass [51], but, nonetheless, identification methods of the study are not adequately discriminatory between *V. alginolyticus* and *V. harveyi*. In this study, *V. alginolyticus* was found mainly during outbreaks in summer and autumn, and it was always considered a co-isolation and not the main cause of disease.

*Photobacterium* spp. are common isolates in aquaculture and wild fish species during the warm months. Incidents of both the subspecies of *P. damselae* causing serious symptoms with gross lesions (including hemorrhages) on almost all of the external body surface parts, turbid eyes, and abdominal distension were studied for wild European seabass and gilthead seabream in the Suez Canal region, with higher prevalence in the first host [52]. However, *P. damselae* subsp. *piscicida* is the causative agent of photobacteriosis (or pasteurellosis, as it is still called by the fish farmers), which is a distinct disease from vibriosis, and although significant for the Mediterranean aquaculture, it is not included in the current study. On the other hand, the second subspecies, *P. damselae* subsp. *damselae*, although genetically similar to the former subspecies, has different phenotypic traits and is considered a causative agent of vibriosis. In our study, this species was always isolated together with *V. harveyi*, making it difficult to assess its clinical significance. *Photobacterium damselae* subsp. *damselae* is considered a primary pathogen for a wide range of aquatic animals and is an emerging pathogen for many aquaculture fish species. More importantly, though, it is a serious human pathogen that has been associated with necrotizing fasciitis, a life-threatening disease, and, therefore, its presence in cultured fish species is alarming [3]. *Photobacterium toruni*, which was identified during a spring outbreak, was first isolated from red-banded seabream (*Pagrus auriga*), and it is a pathogenic species of the genus whose virulent potential is currently explored [53].

Variations of biochemical attributes of *Vibrio* spp. and *Photobacterium* spp. are generally reported because isolates have different phenotypes depending on their host and origin [12,54]. For instance, *Vibrio harveyi* finfish isolates tend to be positive for sorbitol utilization [41,50], contrary to isolates from penaeids and oysters. This was also observed in isolates from gilthead seabream, contrary to isolates from European seabass [40]. This attribute is variable in the isolates included in the current study, not only in *V. harveyi* but also in other vibrios. Variability was observed in several reactions included in the GEN III Microplate, but several demonstrated significant consistency for *V. harveyi*. Almost all isolates tested were positive in the utilization of D-Mannitol, D-Fructose, and Glycerol, and negative in the utilization of D-Melibiose, D-Fucose, Mucic Acid, myo-Inositol, β-Hydroxy-Phenylacetic Acid, Quinic Acid, β-Hydroxy-D,L-Butyric Acid, γ-Amino-Butryric Acid, L-Galactonic Acid Lactone, L-Pyroglutamic Acid, and L-Rhamnose. These reactions could thus be useful traits for the identification of the species. The use of the results of the 94 reactions included in the GEN III Microplate analyzed in this study lead to clustering of the isolates, which reflect the identification of the species.

Vibrios have been associated with climate change and have been proposed as bioindicators of climate change in marine systems [55,56], and since their abundance in the water is temperature related [57], they have rapidly adaptive behavior, more than one portal of entry, and have dominated the temperate climate zone, with outbreaks occurring ever closer to the poles. Thus, it can be speculated that the Mediterranean Sea in the near future will be an ideal setting for more *Vibrio* spp. outbreaks throughout the year.

## 5. Conclusions

Currently, vibriosis is the most significant disease of cultured fish in Greece. Most severe incidences occur during warm months when *Vibrio harveyi* is the most prevalent species, while many different species of the genus are isolated together with *V. harveyi* or with other species during disease outbreaks. The *Vibrio* isolates, included in this study from different hosts and areas, show great variability in their phenotypic traits, pinpointing the importance of molecular identification as a diagnostic routine—a finding that is supported by the intense and long sampling effort of this study.

## Figures and Tables

**Table 1:** Gen III MicroPlate carbon utilization and chemical resistance reaction, with percentages of 73 *V. harveyi* strains shown.

Carbon Utilization	Negative	Positive	Intermediate	Carbon Utilization	Negative	Positive	Intermediate
3-Methyl Glucose	97%	1%	1%	L-Serine	1%	94%	4%
Acetic Acid	7%	81%	13%	Methyl Pyruvate	11%	63%	26%
Acetoacetic Acid	26%	33%	40%	Mucic Acid	100%	0%	0%
Bromo-Succinic Acid	24%	64%	13%	myo-Inositol	97%	0%	3%
Citric Acid	24%	67%	10%	N-Acetyl-Neuraminic Acid	96%	3%	1%
D-Arabitol	68%	14%	18%	N-Acetyl-D-Galactosamine	11%	74%	15%
D-Aspartic Acid	100%	0%	0%	N-Acetyl-D-Glucosamine	1%	92%	7%
D-Cellobiose	3%	85%	13%	N-Acetyl-β-D-Mannosamine	82%	13%	6%
Dextrin	10%	86%	4%	Pectin	7%	85%	8%
D-Fructose	0%	92%	8%	p-Hydroxy-Phenylacetic Acid	100%	0%	0%
D-Fructose-6-PO4	3%	96%	1%	Propionic Acid	81%	11%	8%
D-Fucose	97%	0%	3%	Quinic Acid	100%	0%	0%
D-Galactose	11%	81%	8%	Stachyose	96%	3%	1%
D-Galacturonic Acid	88%	3%	10%	Sucrose	10%	83%	7%
D-Gluconic Acid	1%	93%	6%	Tween 40	10%	63%	28%
D-Glucose-6-PO4	3%	97%	0%	α-D-Glucose	3%	90%	7%
D-Glucuronic Acid	1%	94%	4%	α-D-Lactose	93%	0%	7%
D-Lactic Acid Methyl Ester	86%	3%	11%	α-Hydroxy-Butyric Acid	19%	64%	17%
D-Malic Acid	100%	0%	0%	α-Keto-Butyric Acid	39%	51%	10%
D-Maltose	3%	83%	14%	α-Keto-Glutaric Acid	93%	3%	4%
D-Mannitol	0%	96%	4%	β-Hydroxy-D,L-Butyric Acid	100%	0%	0%
D-Mannose	3%	90%	7%	β-Methyl-D-Glucoside	75%	21%	4%
D-Melibiose	99%	0%	1%	γ-Amino-Butryric Acid	100%	0%	0%
D-Raffinose	96%	0%	4%				
D-Saccharic Acid	99%	1%	0%	**Chemical Resistance**	**Negative**	**Positive**	**Intermediate**
D-Salicin	42%	33%	25%	1% Sodium Lactate	0%	100%	0%
D-Serine	3%	92%	6%	1% NaCl	0%	100%	0%
D-Sorbitol	17%	75%	8%	4% NaCl	0%	100%	0%
D-Trehalose	3%	85%	13%	8% NaCl	0%	100%	0%
D-Turanose	97%	1%	1%	Aztreonam	47%	51%	1%
Formic Acid	99%	1%	0%	D-Serine	1%	99%	0%
Gelatin	29%	56%	15%	Fusidic Acid	0%	100%	0%
Gentiobiose	99%	1%	0%	Guanidine HCl	18%	81%	1%
Glucuronamide	10%	75%	15%	Lincomycin	53%	39%	8%
Glycerol	0%	99%	1%	Lithium Chloride	7%	92%	1%
Glycyl-L-Proline	0%	96%	4%	Minocycline	99%	0%	1%
Inosine	3%	94%	3%	Nalidixic Acid	99%	0%	1%
L-Alanine	0%	97%	3%	Niaproof 4	0%	99%	1%
L-Arginine	44%	42%	14%	pH 5	85%	14%	1%
L-Aspartic Acid	1%	96%	3%	pH 6	0%	100%	0%
L-Fucose	61%	28%	11%	Potassium Tellurite	94%	4%	1%
L-Galactonic Acid Lactone	100%	0%	0%	Rifamycin SV	0%	99%	1%
L-Glutamic Acid	0%	97%	3%	Sodium Broma	75%	25%	0%
L-Histidine	17%	76%	7%	Sodium Butyrate	67%	17%	17%
L-Lactic Acid	0%	97%	3%	Tetrazolium Blue	0%	100%	0%
L-Malic Acid	3%	92%	6%	Tetrazolium Violet	75%	22%	3%
L-Pyroglutamic Acid	100%	0%	0%	Troleandomycin	7%	92%	1%
L-Rhamnose	99%	0%	1%	Vancomycin	3%	97%	0%

## Data Availability

All data have been provided in the article.

## Supplementary Materials

The following supporting information can be downloaded at: https://www.mdpi.com/article/10.3390/microorganisms11051197/s1, Table S1: Primers for PCR and sequencing; Table S2: Identification of bacterial collection; Table S3: mreB sequences downloaded from Genbank.
